# First Principles Study of Adsorption of Hydrogen on Typical Alloying Elements and Inclusions in Molten 2219 Al Alloy

**DOI:** 10.3390/ma10070816

**Published:** 2017-07-19

**Authors:** Yu Liu, Yuanchun Huang, Zhengbing Xiao, Guangze Jia

**Affiliations:** 1 Research Institute of Light Alloy, Central South University, Changsha 410083, China; science@csu.edu.cn (Y.H.); xiaozb@csu.edu.cn (Z.X.); guangzejia@csu.edu.cn (G.J.); 2 Nonferrous Metal Oriented Advanced Structural Materials and Manufacturing Cooperative Innovation Center, Central South University, Changsha 410083, China; 3 College of Mechanical and Electrical Engineering, Central South University, Changsha 410083, China

**Keywords:** first principles, molten 2219 Al alloy, hydrogen, alloying element clusters, inclusions

## Abstract

To better understand the effect of the components of molten 2219 Al alloy on the hydrogen content dissolved in it, the H adsorption on various positions of alloying element clusters of Cu, Mn and Al, as well as the inclusion of Al_2_O_3_, MgO and Al_4_C_3_, were investigated by means of first principles calculation, and the thermodynamic stability of H adsorbed on each possible site was also studied on the basis of formation energy. Results show that the interaction between Al, MgO, Al_4_C_3_ and H atoms is mainly repulsive and energetically unfavorable; a favorable interaction between Cu, Mn, Al_2_O_3_ and H atoms was determined, with H being more likely to be adsorbed on the top of the third atomic layer of Cu(111), the second atomic layer of Mn(111), and the O atom in the third atomic layer of Al_2_O_3_, compared with other sites. It was found that alloying elements Cu and Mn and including Al_2_O_3_ may increase the hydrogen adsorption in the molten 2219 Al alloy with Al_2_O_3_ being the most sensitive component in this regard.

## 1. Introduction

It is well known that the presentation of gases in molten aluminum can cause metallurgy defects [1], such as porosity, in castings, which could raise stress and cause pre-failure in the process of manufacturing and using a product [2]. As reported, hydrogen is the main gas that is dissolved in molten aluminum [3]; when the hydrogen content reaches a critical value in front of metallic solidifying interface, molecular hydrogen bubbles form and may grow, depending on the local hydrogen concentration levels [4]. Hence, the removal of the hydrogen from the molten aluminum prior to casting is crucial for the production of high-quality castings, since it plays a key role in determining the performance of the final products. Several approaches have been developed for degassing, for example, vacuum degassing [5], ultrasonic degassing [6,7], spray degassing [8,9], and rotary impeller degassing with nitrogen, argon, or a mixture of inert gases and using chlorine as a purge gas [10,11].

However, to remove hydrogen from molten aluminum, the behavior of hydrogen in molten aluminum, which is highly associated with the interaction between hydrogen and the components of molten aluminum, must firstly be understood [9,12], since it plays an important role in the process of removing hydrogen, especially for high-efficiency degassing. 

Li studied the effects of alloying the elements Mg and Si on the hydrogen content in molten aluminum. With Hyscan-II hydrogen testing equipment, they found that both Mg and Si elements remarkably enhance the gas-absorbing tendency of molten aluminum [13]. Hu studied the influence of elemental iron on the hydrogen content in superheated molten aluminum-iron alloys experimentally [14], his work indicates that the alloying element iron plays an important role in the hydrogen content of superheated molten Al-Fe alloys below about 1053 K, and that the hydrogen content in molten aluminum reduces with increasing element levels. Li Xizhen studied the influence of the alloying element Cu on the hydrogen content in superheated molten aluminum [15], and findings indicated that the alloying element Cu plays a critical role for the hydrogen content in superheated molten Al-Cu below 780 °C, and that hydrogen content in molten Al decreases with increasing the addition of the alloying element Cu at the same superheated rate. Anyalebechi analyzed hydrogen solubility in liquid Al-H and Al-H-X (where X = Cu, Si, Zn, Fe, Mg, Ti, or Li) alloys via Wagner’s interaction parameter [16], finding that isothermal hydrogen solubility in liquid Al alloys at 101.3 kPa hydrogen partial pressure decreases with an increase of Cu, Si, Zn and Fe levels, but increases with the addition and increase of Mg, Li, and Ti; meanwhile, he found that hydrogen solubility increases with increasing temperature. M. V. Pikunov investigated the effect of the inclusion of aluminum oxide on the processes of hydrogen dissolution and evolution in molten aluminum and copper experimentally [17]; his work explained the relationship between the contamination of molten aluminums by oxide inclusion and porosity of cast billets caused by the evolution of hydrogen bubbles.

Nevertheless, owing to the limitation of experimental equipment and technology, those published works only focused on the macroscopic rules of hydrogen behaviors in molten Al; accordingly, the mechanism is still not completely understood. To our knowledge, few systematic and theoretical studies regarding the interaction between hydrogen and alloying element atom clusters and inclusions in molten aluminum have been reported, while determining the interaction between hydrogen and the molten component is helpful for the design and optimization of degassing technology.

Hence, the present paper investigated the adsorption of H on surfaces of typical inclusion, and alloying element atom clusters of molten 2219 aluminum alloy via first-principles calculations for its high reliability and accuracy, which is a typical Al-Cu-Mn alloy with high fracture toughness and resistant to stress corrosion cracking, and which is widely used in supersonic aircraft skin and structural members. Based on the results obtained, the interaction between hydrogen and the molten 2219 aluminum alloy component is discussed. Accordingly, the alloying elements of Cu and Mn, the most common oxides of Al_2_O_3_ and MgO, and the carbide of Al_4_C_3_, were each considered in present work. 

## 2. Computational Methods and Models

### 2.1. Computational Methods

The Cambridge Sequential Total Energy Package (CASTEP, Accelrys, CA, USA), was used for the first-principles calculations [18]. The ultrasoft pseudopotentials were used to describe the interaction between ions and electrons [19]. General gradient approximation (GGA) of Perdew-Burke-Ernzerhof (PBE) was used to describe the exchange correlation functional, convergence tolerance of total energy per atom is 2 × 10^−6^ eV, and the Brodygen-Fletcher-Gplldfarb-Shanno (BFGS) method was applied for geometry optimization [20]. 

### 2.2. Computational Models

The present studies of H adsorption are focused on close-packed surfaces for their lower energy characteristic [21]; i.e., (111) of Cu and Mn, (0001) of Al_2_O_3_ and Al_4_C_3_, (111) of MgO; meanwhile, the (111) of Al was also calculated for comparisons. These surface models were built based on the optimized bulk structures, and the convergence criteria for structure optimization of SCF tolerance, energy tolerance, maximum force tolerance and maximum displacement tolerance were set to 2.0 × 10^−6^ eV/atom, 2.0 × 10^−3^ eV/atom, 0.02 eV/Å and 5.0 × 10^−4^ Å, respectively. The obtained lattice parameters agreed well with the experimental values [20,22,23,24,25,26], i.e., a = 4.021 for Al, a = 3.641 for Cu, a = 3.594 for Mn, a = 4.806, c = 13.119 for Al_2_O_3_, a = 4.29 for MgO, a = 3.350, c = 25.105 for Al_4_C_3_. 

As for the determination of slab thickness, the convergence tests of Al(111), Cu(111) and Mn(111) surfaces with 3, 5, 7, 9, and 11 layers were conducted with the method proposed in Ref. [27]; accordingly, the energy difference between the slab with the layers of N and N-2 was computed, the results are listed in Table 1. As can be seen from Table 2 that the δE converged when slab thickness increased to 7 layers for all three slabs. Thus, Al(111), Cu(111) and Mn(111) slabs with 7 atomic layers, and a vacuum thickness of 2.7, 2.7 and 1.95 nm, respectively, were adopted in the following calculations. 

Due to the limited space, MgO(111) will be used as an example to demonstrate the convergence test of slab thickness. The change of the interlayer spacing as a percentage of the spacing in the bulk was calculated after full relaxation, as Table 2 shows. It can be seen from the table that the relaxation effects are mainly focused on the top four atomic layers in both termination surfaces; when the slab thickness is more than nine, the top four interlayer relaxations for both termination surfaces are all well-converged, which indicates that slabs with more than nine atomic layers possess a bulk-like interior. Additionally, the outermost interlayer distances of Mg-termination and O-termination surfaces with nine atomic layers are changed by 4.01% and −16.0% of those in the bulk, respectively. Furthermore, the change of the interlayer spacing in the bulk of O-termination surfaces are all larger than those in Mg-termination bulk. Thus, the Mg-termination surfaces are likely to be more stable than O-termination surfaces [32]. Therefore, the following calculation of MgO(111) are based on the slab with nine atomic layers. 

The structure of Al_2_O_3_ in (0001) direction has a O-Al-Al-O-Al-Al-O stacking sequence, so there are three surface terminations (O-I, Al-I, Al-II) when cleaving the bulk structure from the (0001) direction [33]. Similarly, through the convergence test for the slab thickness, Al_2_O_3_(0001) surfaces were modeled with a slab of fourteen atomic layers for the Al-I terminated surface, Al_4_C_3_(0001) surfaces were modeled with a slab of fourteen atomic layers for the Al terminated surface, and the results agree well with the literature [34,35]. The vacuum depth separating the slabs were all set to 1.5 nm. 

**Figure 1:**
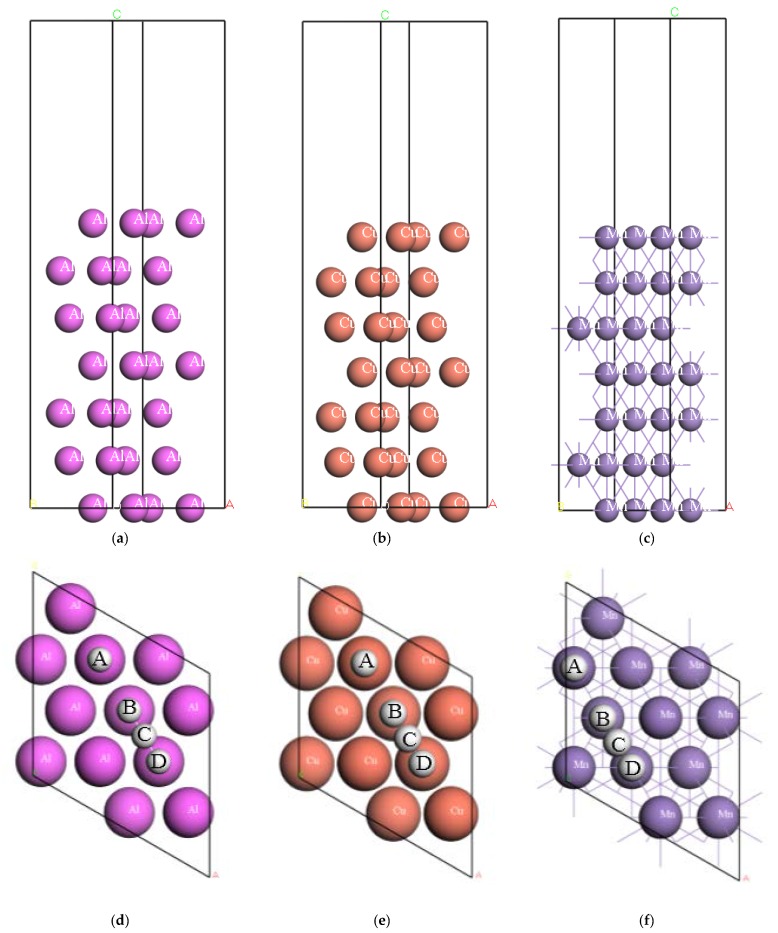
Schematic illustration of the side structure of surface model of (**a**) Al(111), (**b**) Cu(111) and (**c**) Mn(111), along with the adsorption sites of H on (**d**) Al(111), (**e**) Cu(111) and (**f**) Mn(111).

All the surfaces were modeled with a 2 × 2 slab geometry to ensure accuracy. Figure 1 shows the computed models of Al(111), Cu(111) and Mn(111), along with the schematic illustrations of various adsorption positions of H on each surface, where position A, B and D are on the top of the first, third and second atomic layer, respectively, and position C is on the bridge of the first atomic layer. 

**Figure 2:**
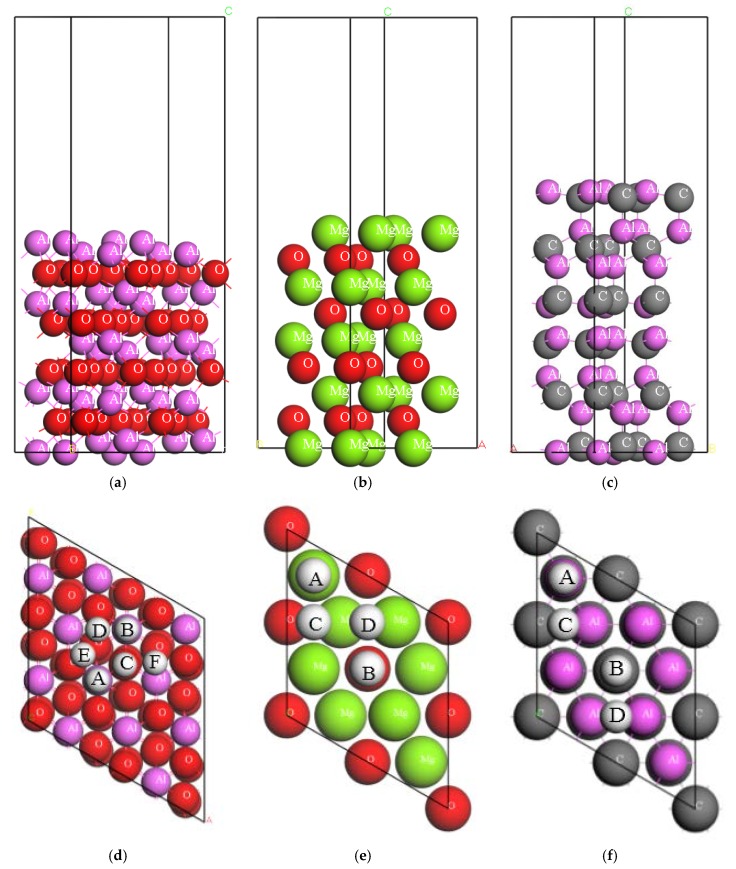
Schematic illustration of the side structure of surface model of (**a**) Al_2_O_3_(0001), (**b**) MgO(111) and (**c**) Al_4_C_3_(0001), along with the adsorption sites of H on (**d**) Al_2_O_3_(0001), (**e**) MgO(111) and (**f**) Al_4_C_3_(0001).

Figure 2 shows the employed models of Al_2_O_3_(0001), Al_4_C_3_(0001) and MgO(111), along with the schematic illustrations of various adsorption positions of H on each surface. As for Al_2_O_3_, we computed six possible adsorption sites of H, i.e., the letters A, B, C, D, E and F in Figure 2, where position A and B are on the top of the Al atom in the first and second atomic layer of the slab, position C is on the top of the O atom in the third atomic layer of the slab, positions D and E are on the bridge of two Al atoms in the first and second atomic layer of the slab, respectively, and position F is on the bridge of two O atoms in the third atomic layer of the slab. Meanwhile, four possible adsorption sites of H on MgO(111) and Al4C3(0001) were studied in the present work. The real position is also demonstrated in Figure 2, where position A, B and D are on the top of the first, second and third atomic layer, respectively, and position C is on the bridge of the first atomic layer. 

In order to balance the accuracy and computation ability, the k-point sampling and plane-wave cutoff energy were determined by testing calculation, Table 3 shows the Atomic layers, total number of atoms, cutoff energy and Kpoints of Al(111), Cu(111), Mn(111), Al_2_O_3_(0001), MgO(111) and Al_4_C_3_(0001), respectively.

## 3. Results and Discussion

### 3.1. Adsorption Energy

The adsorption energy (*E*_ads_) of H atoms on each surface was calculated by the following formula [34,36,37,38]:

*E_ads_* = *E_H/slab_* − *E_slab_* − *E*_*H*2_/2 (1)

where *E_H_*_/*slab*_ and *E_slab_* are the total energies of the slab with and without *H* adsorption, respectively, and *E_H_*_2_ is the total energy of a *H*_2_ molecule.

After a series of calculations, adsorption energy of hydrogen on Cu(111), Mn(111) and Al(111) are listed in Table 4. As we can see, all the *E_ads_* of H adsorbed on Cu(111) and Mn(111) are negative, while the values for Al(111) are all positive, revealing that the interaction between Al and H atoms are mainly repulsive and energetically unfavorable. The calculated results agreed well with other works in the case of Cu(111) and Al(111). Moreover, the *E_ads_* of H adsorbed on site B of Cu(111) and site D of Mn(111) are −2.385 eV and −2.423 eV, respectively, which is lower than that of other sites, meaning that H is more likely to be adsorbed on the top of the third atomic layer of Cu(111) and the second atomic layer of Mn(111), compared with other sites. 

Similarly, the adsorption energy of H on Al_2_O_3_(0001), MgO(111) and Al_4_C_3_(0001) was calculated, and the results are listed in Table 5, from which several highlights can be deduced. Firstly, the *E_ads_* of *H* adsorbed on MgO(111) and Al_4_C_3_(0001) are positive, while the values for Al_2_O_3_(0001) are negative, indicating a favorable interaction between Al_2_O_3_ and H atoms, and that the adatom cannot stably adsorb at MgO(111) and Al_4_C_3_(0001). Secondly, the results obtained for H adsorbed on MgO(111) and Al_2_O_3_(0001) agreed well with the reported data. Thirdly, as for H adsorbed on Al_2_O_3_(0001), three possible sites were determined for H adsorption, and position C, on the top of O atom in the third atomic layer of the slab, was calculated to be the most stable adsorption position for H due to its low *E_ads_* of −2.820 eV, followed by position A and B, on top of the Al atom in the first and second atomic layer of the slab, respectively. 

**Figure 3:**
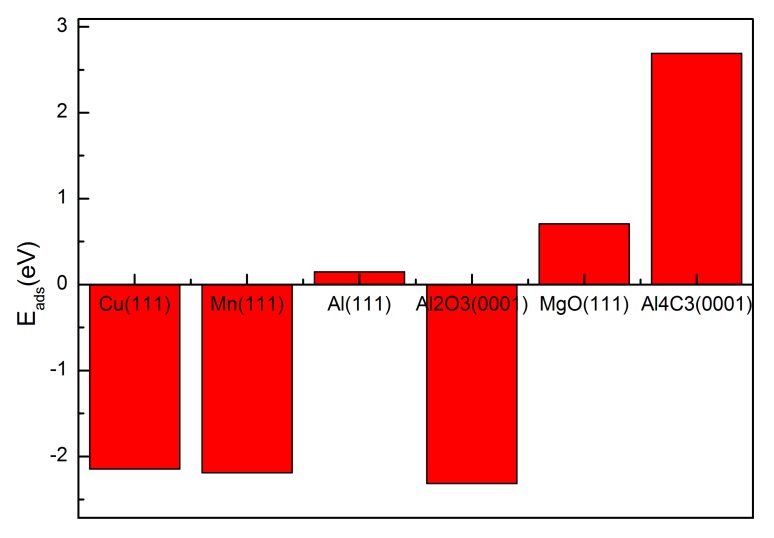
Average adsorption energy of H on Cu(111), Mn(111), Al(111), Al_2_O_3_(0001), MgO(111) and Al_4_C_3_(0001).

Based on the calculations above, the average adsorption energy of H on each surface, as Figure 3 shows, was computed. In general, a negative adsorption energy indicates a tendency to form an adsorption state; hence, we can conclude that alloying elements Cu and Mn and including Al_2_O_3_ may increase the hydrogen adsorption of the molten 2219 aluminum, and Al_2_O_3_ is the most sensitive component in this regard. While MgO and Al_4_C_3_ have little impact on hydrogen concentration in the molten aluminum owing to its weak affinity for hydrogen, especially Al_4_C_3_.

Although references to support the calculation of H adsorbed on Mn(111) and Al_4_C_3_(0001) are still missing, taking account of the computation of other systems, we believe our calculation is reliable and could serve as a reference for future works.

### 3.2. Stability for H Adsorption

Meanwhile, the thermodynamic stability for H adsorption on each surface was studied by calculating the formation energy of H/slab system according to the following formula, which is deduced from Refs. [30,35,41,42]:

*E_f_* = *E_H/slab_* − *E_slab_* − *n_H_μ_H_* (2)

where *n_H_* is the number of the *H* adatoms and *μ_H_* represents the chemical potential of *H* atom. 

In this paper, the chemical potential of H in H_2_O is used as a reference for the chemical potential of hydrogen [43]. According to the conservation of chemical potential in the process of $\frac{1}{2}O_{2}+H_{2}↔H_{2}O$, there exist $\frac{1}{2}\mu_{O_{2}}^{\mathrm{gas}}+\mu_{H_{2}}^{\mathrm{gas}}+\Delta H_{f}^{H_{2}O}=\mu_{H_{2}O}^{\mathrm{molecule}}$. Meanwhile, based on the conservation of chemical potential in a H_2_O molecule, there exist $\mu_{H_{2}O}^{\mathrm{molecule}}=\mu_{O}^{H_{2}O}+2\mu_{H}^{H_{2}O}$, where $\mu_{O_{2}}^{\mathrm{gas}}$ and $\mu_{H_{2}}^{\mathrm{gas}}$ are chemical potential of O_2_ and H_2_, respectively. Accordingly, the calculation of formation energy as a function of the difference of chemical potentials,$\;\Delta \mu_{H}=\mu_{H}^{H_{2}O}-\frac{1}{2}\mu_{H_{2}}^{\mathrm{gas}}$, was performed. 

**Figure 4:**
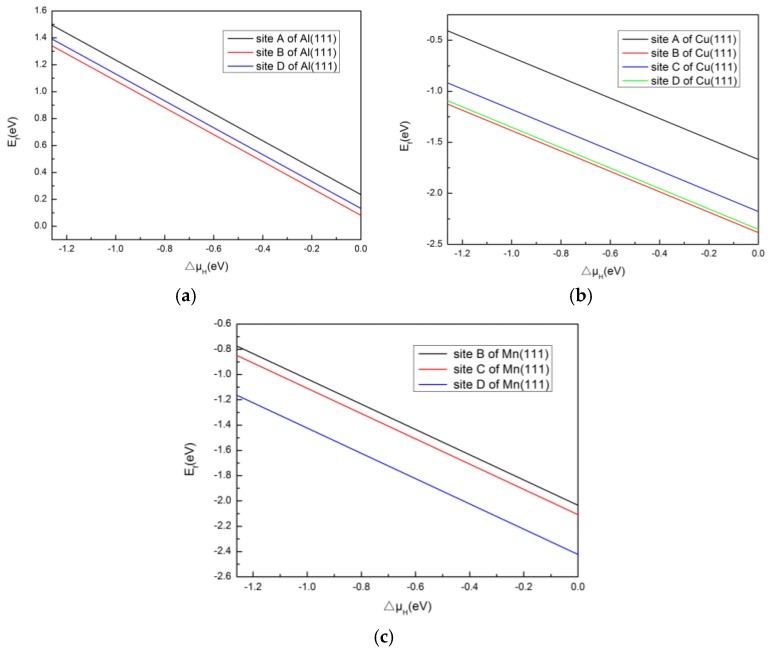
Formation energy of H adsorption on (**a**) Al(111), (**b**) Cu(111) and (**c**) Mn(111) as function of the relative chemical potential of H.

Obviously, negative formation energy indicates a tendency to form a stable structure. Formation energies of H adsorption on Al(111), Cu(111) and Mn(111) as function of the relative chemical potential of H are presented in Figure 4. As can be seem from Figure 4, the formation energy of the H/Mn(111) system is lower than that of H/Al(111) and H/Cu(111), and the formation energies of H/Al(111) were all calculated to be positive, which indicates that it is difficult to form the structure. Additionally, the structure of H adsorption at site B of Al(111) and Cu(111), and site D of Mn(111) is more stable than at other sites for each surface, owing to lower formation energy. This phenomenon indicates that H may tend to adsorb on Mn(111) surfaces, followed by Cu(111), for the main alloying elements in 2219 aluminum alloy.

**Figure 5:**
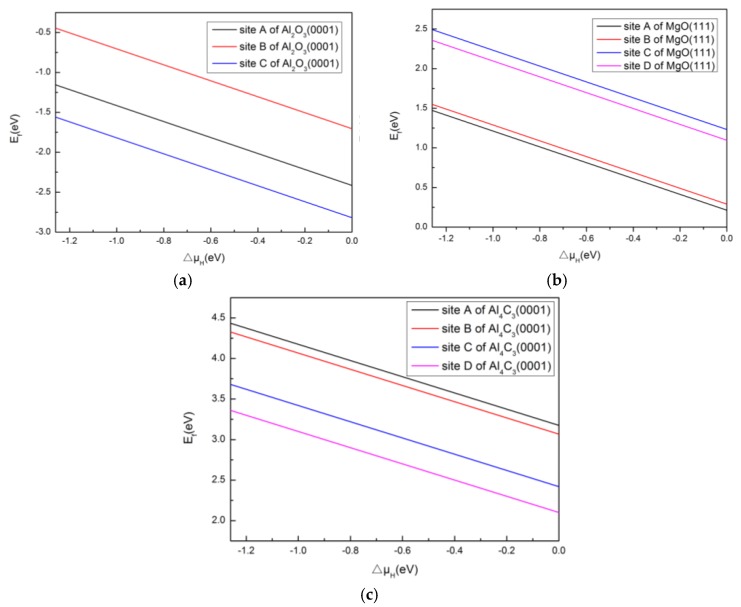
Formation energy of H adsorption on (**a**) Al_2_O_3_(0001), (**b**) MgO(111) and (**c**) Al_4_C_3_(0001) as function of the relative chemical potential of H.

Figure 5 shows the formation energy of H adsorption on Al_2_O_3_(0001), MgO(111) and Al_4_C_3_(0001) as a function of the relative chemical potential of H. As can be seen from Figure 5, the formation energy of H adsorbed on MgO(111) and Al_4_C_3_(0001) were calculated to be positive, which indicates that it is difficult to form the aforementioned structure. The only negative formation energy determined was for H adsorbed on Al_2_O_3_(0001), and site C was the most stable position for H adsorption on Al_2_O_3_(0001), owing to its having the lowest formation energy.

With regard to adsorption energy and formation energy for H adsorption, alloying elements Cu and Mn may increase hydrogen adsorption in molten 2219 aluminum, since they provide more positions for H than Al. As for inclusions of Al_2_O_3_, MgO and Al_4_C_3_, only Al_2_O_3_ can increase hydrogen adsorption, while MgO and Al_4_C_3_ have little impact on hydrogen concentration in the molten aluminum. Moreover, Al_2_O_3_ contributes more to hydrogen content than Cu and Mn in molten 2219 aluminum. 

## 4. Conclusions

In this paper, the interaction between hydrogen and the components of molten 2219 aluminum has been studied. Thus, the H adsorption on various positions of alloying element clusters of Cu, Mn and Al, as well as inclusions of Al_2_O_3_, MgO and Al_4_C_3_, were investigated via first principles calculation, and the thermodynamic stability of H adsorbed on each possible site was also studied on the basis of formation energy. The conclusions are summarized as follows:

(1) The interaction between Al, MgO, Al_4_C_3_ and H atoms is mainly repulsive and energetically unfavorable; a favorable interaction between Cu, Mn, Al_2_O_3_ and H atoms was determined.
(2) H is more likely to be adsorbed on the top of the third atomic layer of Cu(111), the second atomic layer of Mn(111), and the O atom in the third atomic layer of Al_2_O_3_ than other sites.
(3) Alloying elements Cu and Mn and inclusion of Al_2_O_3_ may increase the hydrogen adsorption of the molten 2219 aluminum, and Al_2_O_3_ is the most sensitive component in this regard.

## Figures and Tables

**Table 1:** The energy difference δE of Al(111), Cu(111) and Mn(111) slab.

δE (eV)	5–3	7–5	9–7	11–9
Al(111)	−112.9304	−112.9263	−112.8917	−112.8889
Cu(111)	−2956.4298	−2956.4551	−2956.4633	−2956.4638
Mn(111)	−1310.4996	−1310.4432	−1310.4095	−1310.3995

Al_2_O_3_(0001), MgO(111) and Al_4_C_3_(0001) surfaces are all classified as polar surfaces, because only one species of atoms was present in the terminal surface layer; hence, the convergence tests of those surfaces were conducted with the method proposed in Refs. [28,29,30], and in order to eliminate spurious dipole effects, the symmetric slabs were employed [31].

**Table 2:** Change of interlayer spacing as a percentage of the spacing in the bulk for MgO(111) free surface relaxation.

Slab Thickness												
Interlayer	Mg-Terminated						O-Terminated					
	3	5	7	9	11	13	3	5	7	9	11	13
△_1–2_	−0.60	2.88	10.84	4.01	4.22	4.25	−3.04	−9.1	−12.7	−16.0	−13.4	−13.7
△_2–3_		−2.65	−2.31	−2.35	−2.39	−2.38		6.92	9.35	8.45	8.19	8.26
△_3–4_			−0.42	−0.061	−0.041	−0.043			−2.7	−6.0	−3.4	−3.7
△_4–5_				−0.484	−0.476	−0.478				2.85	2.92	2.94
△_5–6_					−0.323	−0.325					−1.03	−1.06
△_6–7_						0.11						0.32

**Table 3:** Atomic layers, total number of atoms, cutoff energy and Kpoints of Al(111), Cu(111), Mn(111), Al_2_O_3_(0001), MgO(111) and Al_4_C_3_(0001).

Surfaces	Atomic Layers	Total Number of Atoms	Cutoff Energy (eV)	Kpoints
Al(111)	7	7	450	17 × 17 × 1
Cu(111)	7	7	450	21 × 21 × 1
Mn(111)	7	7	430	12 × 12 × 1
Al_2_O_3_(0001)	14	88	350	3 × 3 × 1
MgO(111)	9	36	340	5 × 5 × 1
Al_4_C_3_(0001)	14	56	340	4 × 4 × 1

**Table 4:** Adsorption energy of hydrogen on Cu(111), Mn(111) and Al(111).

Surfaces	Sites	Adsorption Energy (eV)	
		Present Work	Other Works
Cu(111)	A	−1.667	−1.83 [39]
	B	−2.385	−2.37 [39]
	C	−2.178	−2.22 [39]
	D	−2.353	−2.36 [39]
Mn(111)	A	–	–
	B	−2.034	–
	C	−2.109	–
	D	−2.423	–
Al(111)	A	0.235	0.226 [38]
	B	0.081	0.069 [38]
	C	–	–
	D	0.132	0.122 [38]

**Table 5:** Adsorption energy of hydrogen on Al_2_O_3_(0001), MgO(111) and Al_4_C_3_(0001).

Surfaces	Sites	Adsorption Energy (eV)	
		Present Work	Other Works
Al_2_O_3_(0001)	A	−2.416	−2.18 [34]
	B	−1.705	–
	C	−2.820	−2.35 [34]
	D	–	–
	E	–	–
	F	–	–
MgO(111)	A	0.213	0.280 [40]
	B	0.289	0.376 [40]
	C	1.232	–
	D	1.096	0.922 [40]
Al_4_C_3_(0001)	A	3.174	–
	B	3.067	–
	C	2.419	–
	D	2.10	–
